# P-250. Outcomes of Kidney Transplant Recipients among Persons with HIV at the Medical University of South Carolina

**DOI:** 10.1093/ofid/ofaf695.472

**Published:** 2026-01-11

**Authors:** Dhriti K Shah, Jillian S Catalano, Alexandra G Mills, Yosra Alkabab, Courtney E Harris, Ruth O Adekunle

**Affiliations:** Medical University of South Carolina, Charleston, SC; Medical University of South Carolina, Charleston, SC; Medical University of South Carolina, Charleston, SC; MUSC, Charleston, South Carolina; Medical University of South Carolina, Charleston, SC; Medical University of South Carolina, Charleston, SC

## Abstract

**Background:**

Persons with HIV (PWH) are at an increased risk of developing end-stage renal disease (ESRD) compared to individuals without HIV. Kidney transplant is the preferred treatment for ESRD among all patients. Though graft survival between PWH and HIV-negative patients is similar, PWH experience higher rates of rejection. This study describes kidney transplant outcomes among PWH who received post-kidney transplant care at the Medical University of South Carolina (MUSC).

**Figure:**
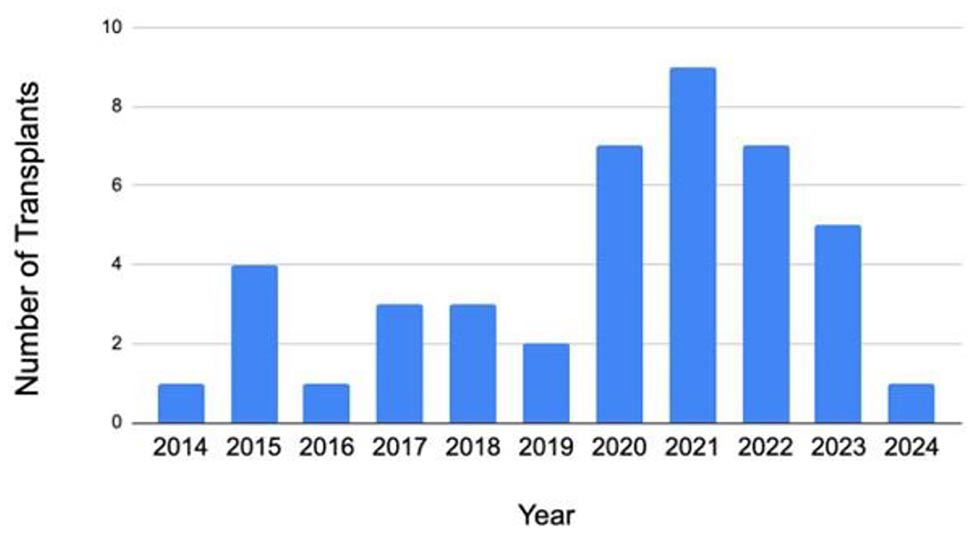
Frequency of PWH receiving a kidney transplant per year

**Methods:**

This was a retrospective review of PWH who received post-kidney transplant care at MUSC Health Care System between May 1st, 2012 and December 31^st^ 2024. Cases were included if sufficient data on their post-transplant care was available in the medical record system. Descriptive statistics were used to analyze clinical characteristics and variables related to post-transplant outcomes.

**Results:**

Forty-four HIV-positive kidney transplant recipients were included, of which, 36 (82%) were transplanted at MUSC. Figure 1 shows kidney transplants performed yearly among PWH. Three (7%) received a living-donor kidney and 2 (5%) received multi-organ transplants. All recipients transplanted at MUSC received anti-thymocyte globulin (ATG) for induction therapy. Twelve recipients (27%) experienced delayed graft function, and 4 (9%) developed graft failure. Rejection was treated in 8 (18%) recipients; the median calculated Panel Reactive Antibody was 38%. Median length of time between transplant and rejection was 1.1 years. Infectious complications included cytomegaloviral disease in 7 recipients (16%), other viral diseases (mostly BK Viremia) in 12 (27%) recipients, and bacterial complications (bacteremia and urinary tract infections) in 6 (14%) recipients. Patient death occurred in 7 patients (16%), with median time to death being 3.3 years. None of the recipients experienced HIV-related complications.

**Conclusion:**

One and three-year graft survival among PWH who received post-kidney transplant care at MUSC was comparable to rates described in the literature. In our study, rejection rates were lower than in the literature, though infectious complications were higher, possibly secondary to universal use of ATG. Managing risk of rejection while minimizing infectious complications remains a challenge.

**Disclosures:**

All Authors: No reported disclosures

